# Merging science and art through fungi

**DOI:** 10.1186/s40694-019-0068-7

**Published:** 2019-04-26

**Authors:** Vera Meyer

**Affiliations:** Department of Applied and Molecular Microbiology, Institute of Biotechnology, Technische Universität Berlin, Gustav-Meyer-Allee 25, 13355 Berlin, Germany

## Abstract

Science and art have long been studied interchangeably, with notable polymaths emerging in the Renaissance such as Leonardo da Vinci (artist, inventor, engineer and anatomist) and Alexander von Humboldt (explorer, geographer and naturalist) with his fellow investigators Johann Wolfgang von Goethe (scientist and writer) and Friedrich Schiller (philosopher, physician and historian). However, this polymathic attitude and the co-operation between scientists and artists seemed to go into hibernation in the second half of the eighteenth century due to an overload of information, especially for the scientists. I illustrate here that the two seemingly diverse fields can feed and sustain each other not only from the attitude of how to think about an object, but also how to show this object in a way that may not have been seen before. Ideas and viewpoints gained from looking at an organism artistically can enable a scientist to think “outside the box”, providing insights to reassess earlier scientifically hidebound attitudes.

Once upon a time, science and art were two sides of the same coin. Scholars practiced both. During the Renaissance, Leonardo da Vinci was one of the greatest exponents of this. He was a polymath and worked as a painter, architect, anatomist and engineer. Scientists and artists working at the same time in the same city, such as Antonie van Leeuwenhook (scientist and engineer) and Jan Vermeer (painter) in Delft in the seventeenth century, learnt from each other’s disciplines and recorded their results. It was mutual inspiration. In the next century, Alexander von Humboldt documented his travels with beautiful drawings and discussed his discoveries with the writers Johann Wolfgang van Goethe and Friedrich Schiller. This polymathic attitude and co-operation between scientists and artists seemed to go into hibernation in the second half of the 18th century due to an overload of information, especially for the scientists. Nowadays, scientists and artists (sometimes, as in the case of the author, the same person) are reinvestigating the natural links between the fields that were jealously guarded specialisations previously.

Is separation of science and art a problem? Yes! As C.P. Snow argues: “But at the heart of thought and creation we are letting some of our best chances go by default. The clashing point of two subjects, two disciplines, two cultures—of two galaxies, so far as that goes—ought to produce creative chances. In the history of mental activity that has been where some of the breakthroughs came. The chances are there now. But they are there, as it were, in a vacuum, because those in the two cultures can’t talk to each other” [1]. My vision is that an effective communication and collaboration between scientists and artists can refill this vacuum with life! And I think that fungal bio(technolo)gists can actively contribute to this. Having fungi as a research subject, we have the chance to connect easily to people outside our field. We are all curious about fungi! (And fungi are curious too.) Actually, many of our non-scientific friends are fascinated by fungi. They enjoy the mushroom season each autumn; they disappear into the woods over the weekend and come home laden with baskets full of mushrooms. What they bring home is only the tip of the iceberg, the fruiting body, which is only a miniscule part of the fungal mycelium. During the summertime, I disappear into my studio and work on paintings, drawings and sculptures. I am then “V. meer” (the attentive reader immediately recognises the genesis of this name: Vera Meyer//Vermeer//V. meer).

I only realized about 5 years ago—because of my dual interest in art and science—that artists and designers have studied Basidiomycota for the last 10 years as both new sustainable producers of composite materials, textiles and leather and efficient decomposers and detoxifiers of our bodies after we have died [2, 3]. These artists and designers were inspired by the Janus-faced head of fungi: fungi are beautiful and morbid; fungi are all-rounders, because they can produce and degrade everything. I summarised their breakthroughs together with my former co-worker Nai [2] in an article in 2016, which received overwhelming attention from both scientists and artists. This was not only because the article highlighted many of the artistic visions which were brought to life by founding biotech start-ups (e.g. Coeio, Ecovative, MycoWorks and NEFFA, [2, 3]) but also because of one big surprise: the artists and designers’ innovations were completely below the radar of the scientific community. None of the artists and designers published their data and insights in scientific journals and many scientists were professionally not interested in collaborations with artists. To quote the designer and founder of MUGO, Maurizio Montalti, who sought to collaborate with fungal laboratories: “Of the 50 or so researchers that I contacted, only a handful answered. In some cases, I was told that they were too busy doing important stuff, implicating that my work wasn’t” [2].

However, there are more similarities than differences between scientific and artistic work! Art and science even share the same beliefs. As Samuel Becket said: “Ever tried. Ever failed. No matter. Try Again. Fail again. Fail better.” I have this quote on the wall in both my lab and my studio. Having mentioned my studio, the visual representation of a studio and a lab is also not that different. Both are work spaces full of materials, tools, installations and equipment to run experiments. Finally, both artists and scientists deliberately venture into the public realm. To quote Hannah Arendt: “Humanity is never won in loneliness and never by handing one’s work over to the public. Only if you take your life and person[ality] into the venture of the public realm, will you reach [humanity]” [4].

This inspired me to synchronise my professional life as a biotechnologist and my passion as an artist and I have been doing this for the last 10 years [5]. Wearing my scientific hat, I am often looking at fungal cell factories through the microscope (thank you, Antonie van Leeuwenhook!) and studying fungal morphology and the patterns it makes. With my artistic hat on, I express their beauty in a different manner (Fig. 1). Sometimes it works, sometimes I fail. And then I remember Becket, because the fungal mycelium I wanted to express on canvas turned out to be beautiful fibroblasts. I failed, but, like the phoenix, something came out of the ashes.

**Fig. 1:**
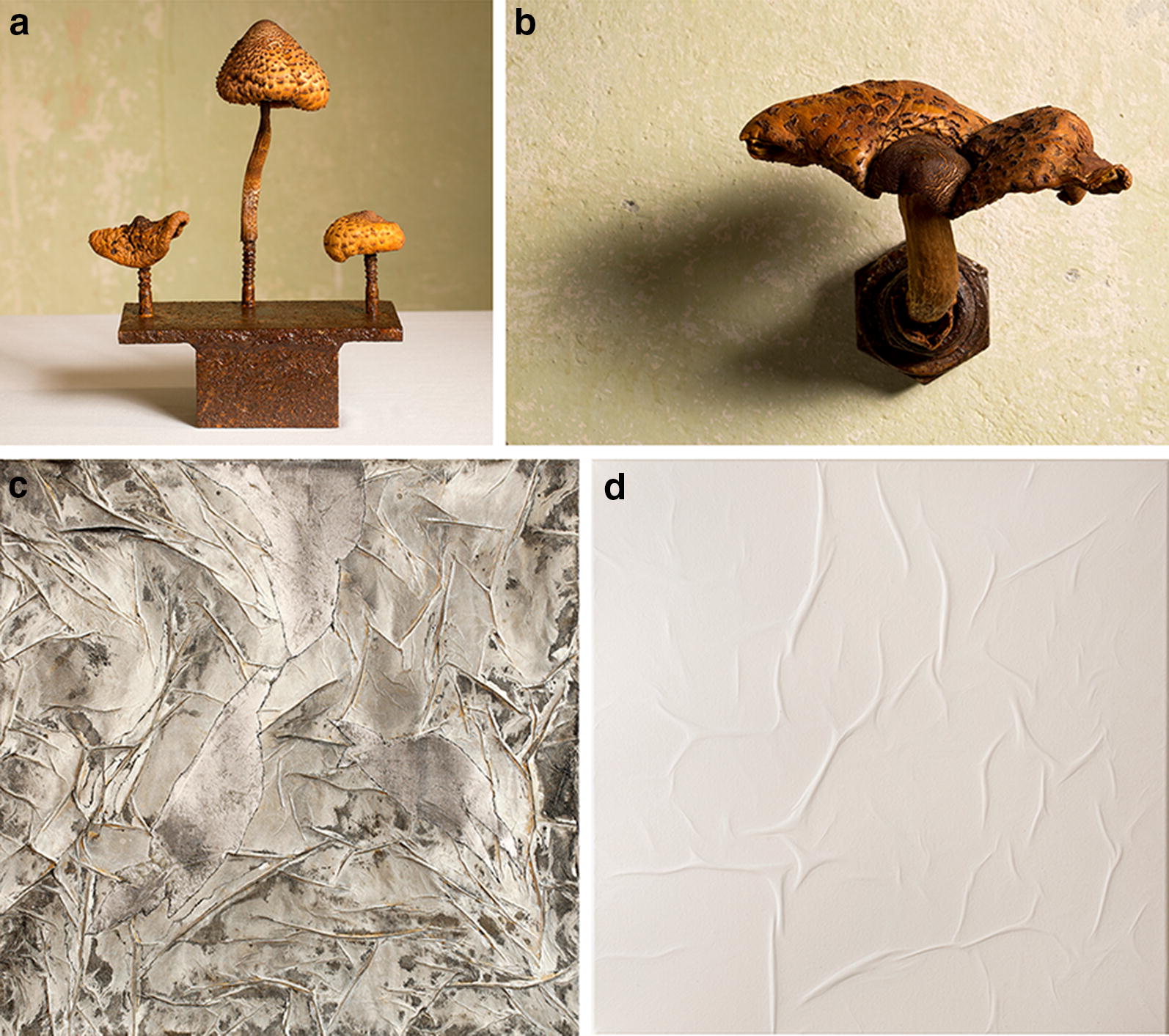
The two seemingly diverse fields biotechnology and art can feed and sustain each other not only from the attitude of how to think about an object, but also how to show this object in a way that may not have been seen before. Four examples are given. **a** “Champi(gn)ons”, 2017. Parasol mushroom, iron stand, shellac, rust, 25 cm × 15 cm × 10 cm. **b** “Spacecraft”, 2017. Parasol mushroom, pipe socket, shellac, rust, 15 cm × 10 cm × 8 cm. **c** “Mycelium”, 2018. Acrylic on canvas, 50 cm × 50 cm. **d** “Fibroblasts”, 2018. Air on canvas, 50 cm × 50 cm. © for all figures: V. meer

I am convinced that the current possibilities in fungal biotechnology have the potential to develop into a disruptive technology. Therefore, early communication and exchange with society is important to us. Successful communication between the sciences and society is particularly important in today’s atmosphere of uncertainty, a lack of factual knowledge and questioning of scientific findings. In my opinion, this can only be achieved through dialogue at eye level. That’s why I initiated the Citizen Science project “Mind the Fungi!” at the Technische Universität Berlin in 2018 and was also able to win over the Art Laboratory Berlin to join it. This research and exhibition platform specialises in co-operation between art, technology and the natural sciences. Thus, jointly organised public lecture series, discussion rounds and workshops between scientists and artists from the Berlin Do-It-Yourself, Bio Art and Citizen Science Communities take place. What is particularly exciting for me is that I can get involved in the project not only as a biotechnologist but also as an artist. I now create sculptures from mushrooms that surprise and (de)mystify, I create a change of perspective through the view of the invisible, through the changed view (looking outside the box) and the changed contextualisation of the visible (Fig. 1).

The philosopher Konrad Paul Liessman once stated: “Art and science are in a tension that is most fruitful when these disciplines observe and penetrate each other and experience how much of the other they themselves still contain” [6]. I fully agree! But perhaps Antonie van Leeuwenhook should have the last word: “… whenever I found out anything remarkable, I have thought it my duty to put down my discovery on paper, so that all ingenious people might be informed thereof” [7]. Was he one of the pioneers of Open Science?
